# 

**Published:** 1891-06-20

**Authors:** 


**PRICE ONE PENNY.**
No. 247, Vol. X.—June 20th, 1891.
[Registered at the General Post Office as a Newspaper for transmission in the United Kingdom and Abroad.]

WITH EXTRA NURSING SUPPLEMENT.
Per Annum, 6s. 6d.; post free.
Monthly Parts, 6d.; post free 8d.
Ditto per Annum, 8s., post free.

### THE HOSPITAL

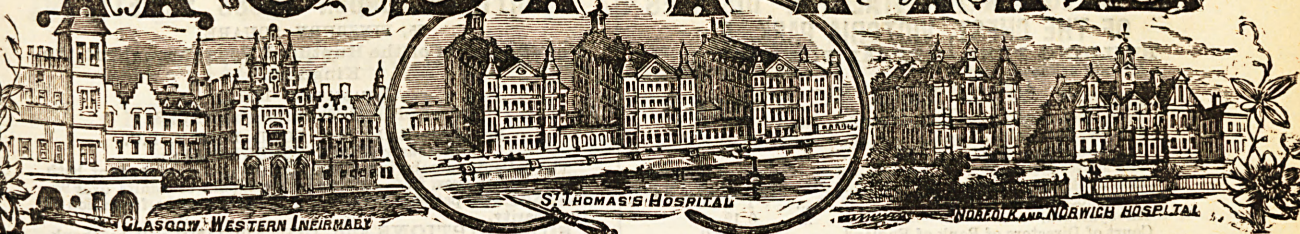

A WEEKLY JOURNAL OF
Science, Medicine, Nursing and Philanthropy.

### SATURDAY, JUNE 20th, 1891.

**The British Pasteur Institute** - 133
**Passing Topics.**—Bazaars and Bankruptcy - 134
**The Doctor's Armchair.**—Literature and Science - 135
**Poison and Poisoners,** III.—Aconitia; Dr. Lamson - 136
**Everybody's Page** - 137
**Notes and News** - 138
**General Charities** - 139
**Scraps and Gleanings** - 139
**Annotations.**—The Poor Clergyman's Holiday—Why Men Gamble—Laundresses on Protest - 140
**The Late Dr. H, G. Sutton** - 144
**The Practitioners' Mirror and Retrospect**—
Medicine.—Syringomyelia.—II., 14 ; Absorption of Fats 142
Surgery.—The Surgery of the Spinal Cord - 142
Therapeutics.—The Action of Caffeine, Morphine, Atropine, Ergot, and Digitalis on the Arterial Blood Pressure - 143
New Drugs, Appliances, and Things Medical—A New Plaster of Paris Bandage Roller—A New Brown Bread 144
**Trained Attendants and Nurses for the Insane** - 144
**The Student of Medicine**—Editorial Notes—Appointments—Vacancies—Scholarships and Prizes—Pass Lists—

### EXTRA SUPPLEMENT—THE NURSING MIRROR.

**For Reading to the Sick**—Where are you Going? - lxvii

**A Year in a German Hospital.**—Part III. - lxviii

**Nurse Training in the Seventies.** By E. D. (*continued*) lxix

**Everybody's Opinion.**—British Nurses Again—The Dulness of Domestic Service - lxix

**Wants and Workers** - lxix

**Off Duty.**—One Evening—**Presentation** - lxx

**Notes and Queries** - lxx

**Amusements and Relaxation** - lxx

**En Passant.**—The Brassey Holiday Home—The Santa Claus Home—The Nurse-Doctor System—St. Mary Abbotts Infirmary—Domestic Service—Short Items—The Advance of the Attendant - lxv

**Lectures on Surgical Ward Work and Nursing,** By Alex. Miles, M.B. Edin., C.M., F.R.C.S.E.—Lecture XXVII.—Bandaging - lxvi

**Women as County Councillors** - lxvii

**Appointments** - lxvii

